# Dr. Peoples’ Miasmatic Paralytic Fever

**Published:** 1895-08

**Authors:** Thomas A. Pope

**Affiliations:** Cameron, Texas


### Correspondence.

### Dr. Peoples' Miasmatic-Paralytic Fever.

CAMERON, TEXAS, July 25, 1895.

*Editor Texas Medical Journal:*

In your July number you ask for comment on the article

"Miasmatic Paralytic Fever," published in the June number of the "RedBack."

I would have replied to this request earlier had it been *possible.* The reason for the delay must be evident to every one who has read the article. The immense erudition displayed rendered several lexicons, some medical, others theological, necessary, but even after patient stndy with these aids, I am not sure that I fully comprehend but a small portion of the article; but I hope before I feel the "relentless clasping of mortuary shackles" to arrive at a clear understanding.

I don't understand whether it is the patient or the physician who is so powerfully affected by the "tertiary;" but in any case, it must be interesting to witness,—not death,—but "immediate translation,"—"Carnality assume its spiritual and eternal form!" I am not surprised that there is a "dearth of literature on this subject in medical works." It is indeed greatly to be regretted that "our best authors have never had a case of miasmatic paralytic fever," and it is indeed, most wonderful that of all the thousands of educated and careful observers living in malarious districts all over the world, not one has recorded such a case.

A Columbus was needed to discover the New World. He was a man among many millions, and so we must regard the discoverers of to-day in the field of medicine, and honor them accordingly.

The author makes several statements that could only be verified by careful and repeated dissection of patients dying with this fever, so I take it for granted he has performed these. I did intend to go all over this article, but I desist.

The doctor has given an exaggerated description of a trouble which every one who practices in malarial countries has repeatedly seen. As I understand him, he was trying to describe a case of pernicious remittent,—not "black jaundice,"—"swamp fever" nor "malaria hemorrhagica," as you suggest.

Yours truly, THOMAS A. POPE.

